# The predictive value of atherogenic index of plasma for cardiovascular outcomes in patients with acute coronary syndrome undergoing percutaneous coronary intervention with LDL-C below 1.8mmol/L

**DOI:** 10.1186/s12933-023-01888-3

**Published:** 2023-06-26

**Authors:** Yue Wang, Shen Wang, Shuaifeng Sun, Fadong Li, Wenxin Zhao, Hongxia Yang, Xiaofan Wu

**Affiliations:** Department of Cardiology, Beijing Anzhen Hospital, Capital Medical University, 2th Anzhen Road, Chaoyang District, Beijing, 100029 China

**Keywords:** Atherogenic index of plasma, Acute coronary syndrome, Percutaneous coronary intervention, LDL-C < 1.8mmol/L

## Abstract

**Background:**

The potential predictive significance of atherogenic index of plasma (AIP) for cardiovascular outcomes in patients with acute coronary syndrome (ACS) and who have undergone percutaneous coronary intervention (PCI), with low-density lipoprotein-cholesterol (LDL-C) below 1.8mmol/L, has not been well explored.

**Methods:**

The retrospective cohort analysis included 1,133 patients with ACS and LDL-C levels below 1.8mmol/L who underwent PCI. AIP is calculated as log (triglyceride/high-density lipoprotein-cholesterol). Patients were divided into two groups according to the median value of AIP. The primary endpoint was major adverse cardiovascular and cerebrovascular events (MACCEs), a composite of all-cause death, nonfatal myocardial infarction, ischemic stroke or unplanned repeat revascularization. The association between AIP and the prevalence of MACCE was evaluated using multivariable Cox proportional hazard models.

**Results:**

Over a median follow-up of 26 months, the incidence of MACCE was higher in the high AIP group compared to the low AIP group (9.6% vs. 6.0%, *P* log-rank = 0.020), and the difference was mainly derived from an increased risk of unplanned repeat revascularization (7.6% vs. 4.6%, P log-rank = 0.028). After adjusting for multiple variables, elevated AIP was independently associated with an increased risk of MACCE, regardless of whether AIP was considered a nominal or continuous variable (hazard ratio [HR] 1.62, 95% confidence interval [CI] 1.04–2.53 or HR 2.01, 95% CI 1.09–3.73).

**Conclusions:**

The present study demonstrates that AIP is a significant predictor of adverse outcomes in ACS patients undergoing PCI with LDL-C < 1.8mmol/L. These results suggest that AIP may offer supplementary prognostic information for ACS patients with optimally managed LDL-C levels.

**Supplementary Information:**

The online version contains supplementary material available at 10.1186/s12933-023-01888-3.

## Introduction

Acute coronary syndrome (ACS) is the most ominous manifestation of coronary artery disease (CAD) and continues to be the leading cause of mortality globally. Despite the significant reduction in cardiovascular events through the rapid development and extensive use of Percutaneous Coronary Intervention (PCI), the prognosis for ACS patients remains unsatisfactory [1]. Dyslipidemia, characterized by elevated levels of low-density lipoprotein-cholesterol (LDL-C), total cholesterol (TC), triglycerides (TG), or reduced levels of high-density lipoprotein-cholesterol (HDL-C), is a prevalent condition among patients with ACS and is an important conventional risk factor for ACS, contributing to a poor prognosis [2]. Current guidelines recommend targeting LDL-C as the primary objective of lipid-lowering interventions in ACS patients [3]. However, despite achieving optimal LDL-C levels through intensified lipid-lowering medication, some patients remain at an increased risk of recurrent cardiovascular events [4], which indicates that it is not sufficient to focus on LDL-C level. There is increasing focus on identifying lipid related residual risk factors for better predicting cardiovascular outcomes and improving clinical management.

LDL-C is comprised of particles that exhibit varying sizes, densities, and properties. It has been demonstrated that the smaller and denser the particles, the more susceptible it is to oxidation and the greater its atherogenic potential [5]. Atherogenic index of plasma (AIP), which is calculated using the formula log (TG/HDL-C), has been proven to be significantly correlated with lipoprotein particle size and density, as well as lipoprotein peroxidation rates, and could be used as a reliable marker of plasma atherogenicity [6, 7]. Numerous studies have suggested that AIP has a strong relationship with the risk of heart failure, stroke and CAD [8–10], and is independently associated with poor prognosis in patients with CAD [11, 12]. However, these studies did not account for the LDL-C level, which was above the recommended threshold, potentially impacting the significance of AIP on cardiovascular prognosis. Whether AIP is still associated with poor prognosis in patients ACS and who have undergone PCI, with LDL-C below 1.8mmol/L remains less well explored. Therefore, this current study aimed to investigate the predictive value of AIP in adverse cardiovascular events in this patient population.

## Method

### Study Population

This is a retrospective, single-center observational study conducted at Beijing Anzhen Hospital, Capital Medical University between January 2017 to May 2019. Among 5,277 patients with ACS who underwent PCI initially screened, 3,548 patients with at least one LDL-C measurements before and during index hospitalization (from 7 days before admission until discharge) and complete baseline data were identified (Table S1). The participants with severe liver or kidney failure, those who suffering PCI failure and in-hospital death, or those who missing follow-up data were excluded.

Ultimately, 1,133 patients with an LDL-C < 1.8mmol/L were included in the present analysis. The procedures were executed in compliance with the Declaration of Helsinki and were approved by the Ethics Committee of Beijing Anzhen Hospital. All the patients provided written informed consent before the index PCI.

### Procedures and treatment

The interventions for coronary revascularization were performed in accordance with the prevailing practice guidelines [13], and were selected left to the operators’ discretion. After the procedure, all patients were recommended to receive optimal pharmacological therapy as per the standard regimen. The dual antiplatelet therapy of aspirin 100 mg once daily and ticagrelor 90 mg twice daily was continued for a minimum of 12 months, with the possibility of extending the ticagrelor treatment period at the operator’s discretion. Patients with high bleeding risk or who underwent complicated procedures were also administered clopidogrel 75 mg once daily or ticagrelor 45 mg twice daily at the discretion of the operator.

### Data collection and definition

Patient medical records were utilized to gather information regarding demographics, clinical characteristics, angiographic and procedural details, and laboratory data. Fasting venous blood samples were collected in the early morning of the day following admission and were expeditiously transported to the core laboratory of Beijing Anzhen Hospital. Serological parameters including TG, TC, LDL-C, HDL-C, fasting plasma glucose (FBG), glycosylated hemoglobin (HbA1c), creatinine, and uric acid were analyzed by standard laboratory methods. AIP was calculated using the following formula ln [log (TG/HDL-C) [6]. The images of coronary angiogram and PCI, such as target vessel territory, the number of stents, minimal stent diameter, and total stent length were analyzed separately by two interventional cardiologists.

Hypertensions was defined as having a previous history of hypertension, systolic blood pressure ≥ 140 mmHg and/or diastolic blood pressure ≥ 90 mmHg, or receiving antihypertensive agents. Diabetes mellitus was defined as having a previous history of diabetes mellitus, HbA1c level ≥ 6.5%, or receiving glucose-lowering therapy. Dyslipidemia was defined as having a definite diagnosis of dyslipidemia, TG ≥ 2.3 mmol/L, LDL-C ≥ 1.8 mmol/L or HDL-C < 1.0 mmol/L, or receiving lipid-lowering agents. The estimated glomerular filtration rate was calculated using the Chronic Kidney Disease Epidemiology Collaboration (CKD-EPI) equation [14].

### Endpoints and follow‑up

The primary endpoint was the occurrence of major adverse cardiovascular and cerebrovascular events (MACCEs), defined as cardiac death, non-fatal myocardial infarction (MI), non-fatal stroke, and unplanned repeat revascularization. Secondary endpoints included all-cause death and all components of the primary endpoint. All deaths were considered of cardiac origin unless a noncardiac origin was established clinically or at autopsy [15]. Non-fatal MI was defined in accordance with the fourth universal definition of MI [16]. Non-fatal stroke is defined as any acute new neurological deficit lasting longer than 24 h accompanied by neuroimaging evidence of brain ischemia or bleeding [17]. Unplanned repeat revascularization was defined as any revascularization driven by angina or ischemia, either PCI or coronary artery bypass graft, of any segment of the target or nontarget vessel [15]. All clinical events were evaluated independently by at least two members of the clinical event committee. The participants were followed-up via telephone calls or outpatient visits until December 31, 2020, or until their demise.

### Statistical analysis

Descriptive statistics are presented in the form of mean ± standard deviation, medians (interquartile range [IQR]) or frequencies (percentages). Differences between groups were compared using the Student’s t-test or Wilcoxon rank sum test for continuous variables, while the chi-square test or Fisher exact test for categorical variables, as appropriate. Participants were categorized based on the median value of AIP or the occurrence of MACCE. The cumulative incidence of endpoint events was depicted using Kaplan-Meier curves, and differences among groups were assessed using the log-rank test. Cox proportional hazard regression models were utilized to estimate the association of AIP with the incidence of MACCE. Three models were built as follows: Model 1, unadjusted model; Model 2, only adjusting age and sex; Model 3, adjusting sex, age, body mass index (BMI), hypertension, dyslipidemia, diabetes mellitus, previous MI, previous stroke, oral hypoglycemic agents, LDL-C, TC, HbA1c, and uric acid. Covariates for the adjusted models were selected based on clinical or statistical significance. In the Cox model, AIP were modeled as categorical or continuous (per 1-unit increase). Additionally, the predictive value of AIP for secondary endpoints was also evaluated by univariate and multivariate Cox proportional hazards analyses adjusting for all the variables in Model 3. These results were reported as hazard ratios (HRs) with 95% confidence intervals (CIs). Further stratified analyses were performed to evaluate the association between AIP and MACCE among the following groups: age (≤ 65 and > 65 years), sex, BMI (≤ 28 and > 28 kg/m^2^), hypertension, HbA1c (≤ 6.5 and > 6.5%), and the type of ACS. utilized Cox proportional hazard Model 3 to employ Restricted Cubic Spline Regression with 4 knots fitted for Cox proportional hazard Model 3 was used to explore the potential linear or nonlinear relations between AIP and MACCE risk. Statistical analyses were performed with SPSS 25.0 (SPSS Inc., Chicago, Illinois, USA) and Stata version 14.0 (Stata Corp., College Station, TX, USA). All p values were 2-tailed, with statistical significance set at < 0.05.

## Results

**Fig. 1:**
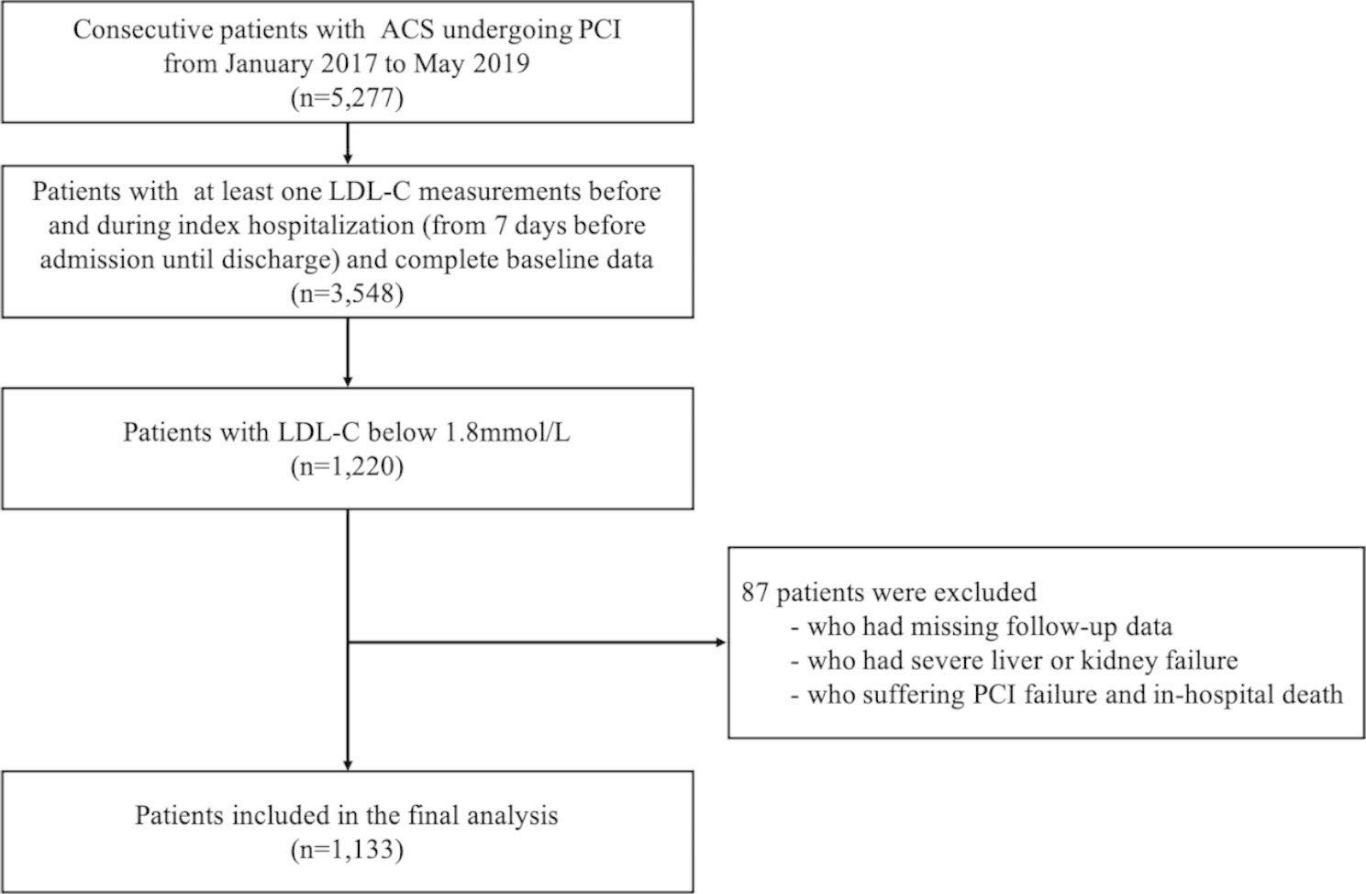
Flow diagram of the study population. ACS, acute coronary syndrome; AIP, atherogenic index of plasma; LDL-C, low-density lipoprotein-cholesterol; PCI, percutaneous coronary intervention

The study cohort finally consisted of 1,133 participants (Fig. 1). The mean age was 58.6 ± 9.5 years, and 966 (85.3%) were men. The mean value of AIP was 0.11 (IQR − 0.07, 0.33), and the frequency histograms of AIP presented in Figure S1. Over a median follow-up of 26 months, 88 cases of MACCEs were recorded, including 12 (1.0%) all-cause deaths (10 from cardiovascular causes), 9 (0.7%) non-fatal MIs, 5 (0.4%) non-fatal strokes, and 69 (6.0%) unplanned repeat revascularizations.

### Baseline characteristics

The baseline characteristics stratified by the median value of AIP were listed in Table 1. Patients who were categorized in the high AIP group were older and more likely to be smokers, were more likely to have experienced dyslipidemia, previous MI, and previous stroke, exhibited much higher BMI, TC, TG, FPG, HbA1c, and uric acid level, and more frequently used oral hypoglycemic agents compared to those in the low AIP group (*P* < 0.05). Table S2 describes baseline characteristics based on the occurrence of MACCE. Compared with those remained MACCE-free, patients who experienced MACCE showed a higher prevalence of previous MI, higher TG and AIP level, and higher medication rates of alpha glucosidase inhibitor (*P* < 0.05).

**Table 1:** Participant characteristics stratified by the median value of AIP

	Lower AIP level (< 0.11; n = 570)	Higher AIP level (≥ 0.11; n = 563)	P value
**Age (y)**	60.6 ± 9.2	56.5 ± 9.4	< 0.001
**Sex, male**	484 (84.9)	482 (85.6)	0.739
**BMI, kg/m** ^**2**^	25.3 ± 2.9	26.5 ± 3.0	< 0.001
**Risk factors**			
Hypertension	350 (61.4)	367 (65.2)	0.187
Dyslipidemia	257 (45.1)	377 (67.0)	< 0.001
Diabetes mellitus	222 (38.9)	263 (46.7)	0.008
Current smoker	201 (35.3)	256 (45.5)	< 0.001
**Medical history**			
Prior MI	123 (21.6)	168 (29.8)	0.001
Prior PCI	196 (34.4)	212 (37.7)	0.252
Prior CABG	8 (1.4)	13 (2.3)	0.258
Prior stroke	42 (7.4)	19 (3.4)	0.003
PAD	6 (1.1)	10 (1.8)	0.302
CKD	10 (1.8)	18 (3.2)	0.118
**ACS type**			0.277
STEMI	43 (7.5)	53 (9.4)	
NSTEMI	43 (7.5)	52 (9.2)	
Unstable angina	484 (84.9)	458 (81.3)	
**Procedure characteristics**			
Lesion complexity			
Left main lesion	76 (13.3)	59 (10.5)	0.138
Bifurcation lesion	72 (12.6)	55 (9.8)	0.127
Chronic total occlusion	133 (23.3)	139 (24.7)	0.593
Target vessel territory			
Left main	53 (9.3)	41 (7.3)	0.219
Left anterior descending artery	347 (60.9)	320 (56.8)	0.167
Left circumflex	155 (27.2)	142 (25.2)	0.451
Right coronary artery	173 (30.4)	214 (38)	0.007
Multivessel intervention	165 (28.9)	161 (28.6)	0.896
Stent number	2 (1.0, 2.0)	2 (1.0, 3.0)	0.468
Mean stent diameter, mm	3 ± 0.5	3 ± 0.4	0.554
Total stent length, mm	37 (1.0, 2.0)	38 (24.0, 63.0)	0.168
**Laboratory results**			
LDL-C, mmol/L	1.5 ± 0.2	1.5 ± 0.2	0.733
HDL-C, mmol/L	1.1 ± 0.2	0.9 ± 0.2	< 0.001
TC, mmol/L	3 ± 0.4	3.2 ± 0.7	< 0.001
TG, mmol/L	0.9 (0.7, 1.1)	1.8 (1.4, 2.7)	< 0.001
FPG, mmol/L	5.81 (5.2, 7.0)	6.2 (5.4, 8.3)	< 0.001
HbA1c, %	6.4 ± 1.2	6.6 ± 1.4	0.001
Creatinine, µmol/L	74.3 ± 38.3	77.9 ± 48	0.155
Uric acid, µmol/L	332.5 ± 80.9	367.4 ± 85	< 0.001
LVEF, %	62.54 ± 6.7	61.7 ± 7.4	0.053
**Medications at discharge**			
Aspirin	569 (99.8)	562 (99.8)	1.000
Ticagrelor	570 (1)	563 (1)	1.000
Statin	566 (99.3)	560 (99.5)	1.000
Ezetimibe	42 (13.3)	39 (11.4)	0.461
β-Blocker	305 (53.5)	327 (58.1)	0.121
ACEI/ARB	295 (51.8)	313 (55.6)	0.195
Calcium-channel antagonist	167 (29.3)	159 (28.2)	0.694
Proton pump inhibitor	515 (90.4)	494 (87.7)	0.16
Oral hypoglycemic agents	100 (17.5)	134 (23.8)	0.009
Metformin	44 (7.7)	71 (12.6)	0.024
Alphaglucosidase inhibitor	64 (11.2)	78 (13.9)	0.396
Meglitinide	12 (2.1)	10 (1.8)	0.744
Sulfonylurea	31 (5.4)	33 (5.9)	0.811
Thiazolidinediones	6 (1.1)	0 (0)	0.012
DPP-4 inhibitor	4 (0.7)	3 (0.5)	0.765
Insulin	32 (5.6)	51 (9.1)	0.083

ACEI/ARB, angiotensin converting enzyme inhibitors/angiotensin receptor blockers; AIP, Atherogenic index of plasma; BMI, body mass index; CABG, coronary artery bypass grafting; CKD, chronic kidney disease; CTO, chronic total occlusion; DPP-4, dipeptidyl peptidase-4; FPG, fasting plasma glucose; HbA1c, glycosylated hemoglobin; HDL-C, high density lipoprotein cholesterol; LDL-C, low density lipoprotein cholesterol; LVEF, left ventricular ejection fraction; MI, myocardial infarction; NSTEMI, No ST-segment elevation myocardial infarction; PAD, peripheral arterial disease; PCI, percutaneous coronary intervention; STEMI, ST-segment elevation myocardial infarction; TC, total cholesterol; TG, triglyceride

### AIP and risk of endpoint events

**Fig. 2:**
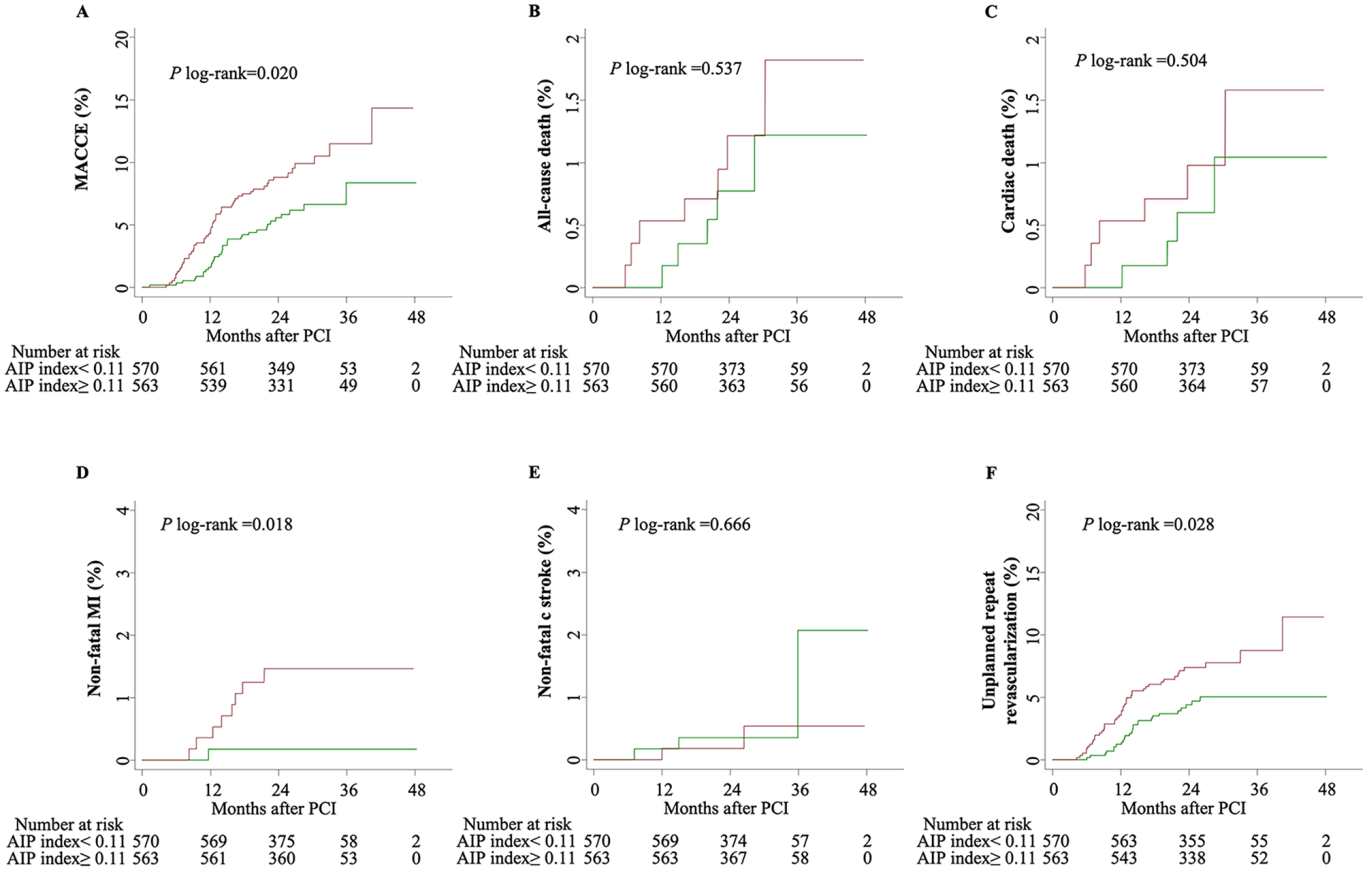
Kaplan-Meier curves for the cardiovascular events based on the median value of AIP. (**A**) MACCE; (**B**) all-cause death; (**C**) cardiac death; (**D)** non-fatal MI; (**E)** non-fatal stroke; (**F)** unplanned repeat revascularization. AIP, atherogenic index of plasma; CI, confidence interval; MACCE, major adverse cardiovascular and cerebrovascular events; MI, myocardial infarction; PCI, percutaneous coronary intervention

**Fig. 3:**
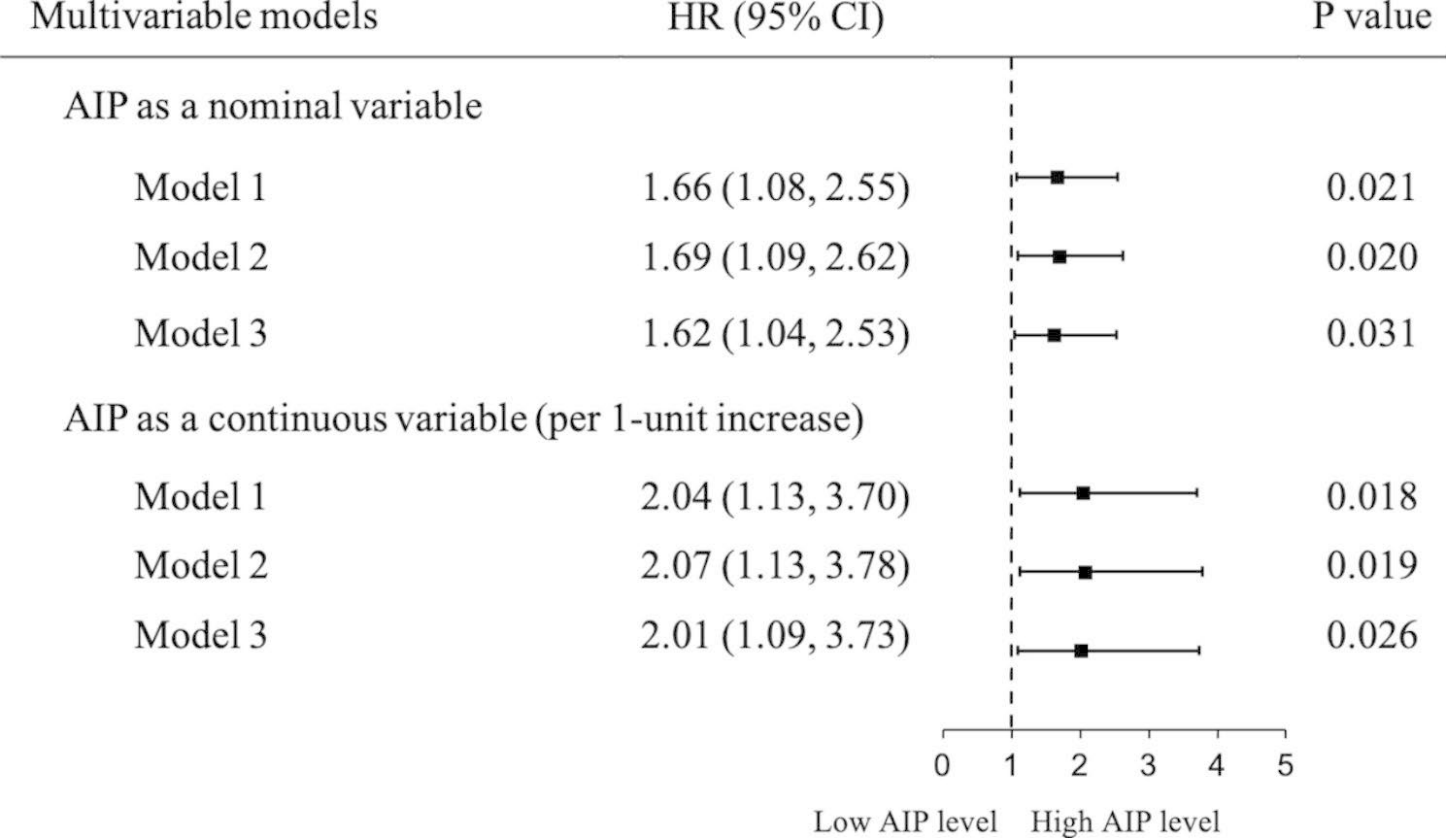
Multiple Cox proportional hazard models for the impact of AIP on the incidence of MACCE. AIP were modeled as categorical or continuous (per 1-unit increase). Model 1 is unadjusted. Model 2 includes age and sex; Model 3 includes the variables in model 2 and body mass index, hypertension, dyslipidemia, diabetes mellitus, previous MI, previous stroke, oral hypoglycemic agents, low-density lipoprotein-cholesterol, total cholesterol, glycosylated hemoglobin, and uric acid. AIP, atherogenic index of plasma; CI, confidence interval; HR, hazard ratio; MACCE, major adverse cardiovascular and cerebrovascular event; MI, myocardial infarction

Figure 2 illustrates Kaplan-Meier curves for endpoint events, categorized by the median value of AIP. Individuals in the high AIP group experienced a higher risk of MACCE and unplanned repeat revascularization than those in the low AIP group (9.6% vs. 6.0%, *P* log-rank = 0.020 and 7.6% vs. 4.6%, *P* log-rank = 0.028). The HRs for MACCE from the three Cox regression models are shown in Fig. 3. The categorical analysis demonstrated that the high AIP level was significantly associated with a higher risk of MACCE compared with the low AIP level (HR 1.66, 95% CI 1.08–2.55), and remained significant even after adjustment for potential confounding factors (in Model 2: HR 1.69, 95% CI 1.09–2.62; in Model 3: HR 1.62, 95% CI 1.04–2.53). The findings remained similar when AIP was incorporated as a continuous variable in the models. The HR for MACCE in subjects with the high AIP group versus the low AIP group was 2.04 (1.13–3.70) in model 1, 2.07 (1.13–3.78) in model 2 and 2.01 (1.09–3.73) in model 3.

The association of AIP with the risk of second endpoint events was also evaluated in the univariate and multivariate Cox regression analysis (Table 2). In the fully adjusted model (model 3), the risk of unplanned repeat revascularization was significantly higher in the high AIP group versus the low AIP group (HR1.74, 95% CI 1.06–2.87), while the difference in other events risks was insignificant between the same groups.

**Table 2:** Association of AIP with the secondary endpoint events

	Low AIP level (< 0.11; n = 570)	High AIP level (≥ 0.11; n = 563)	Univariate analysis			Multivariate analysis	
			HR (95%CI)	P value		HR (95%CI)	P value
All-cause death	5 (0.9)	7 (1.2)	1.43 (0.46, 4.51)	0.539		1.51 (0.47, 4.88)	0.494
Cardiovascular death	4 (0.7)	6 (1.1)	1.53 (0.43, 5.44)	0.507		1.58 (0.43, 5.77)	0.487
Non-fatal MI	1 (0.2)	8 (1.4)	8.14 (1.02, 65.07)	0.048		5.47 (0.67, 44.97)	0.114
Non-fatal stroke	3 (0.5)	2 (0.4)	0.68 (0.11, 4.04)	0.668		0.45 (0.06, 3.25)	0.425
Unplanned revascularization	26 (4.6)	43 (7.6)	1.72 (1.06, 2.80)	0.029		1.74 (1.06, 2.87)	0.029

Multivariate analysis includes age, sex, body mass index, hypertension, dyslipidemia, diabetes mellitus, previous MI, previous stroke, oral hypoglycemic agents, low-density lipoprotein-cholesterol, total cholesterol, glycosylated hemoglobin, and uric acid. AIP, atherogenic index of plasma; CI, confidence interval; MI, myocardial infarction; HR, hazard ratio

**Fig. 4:**
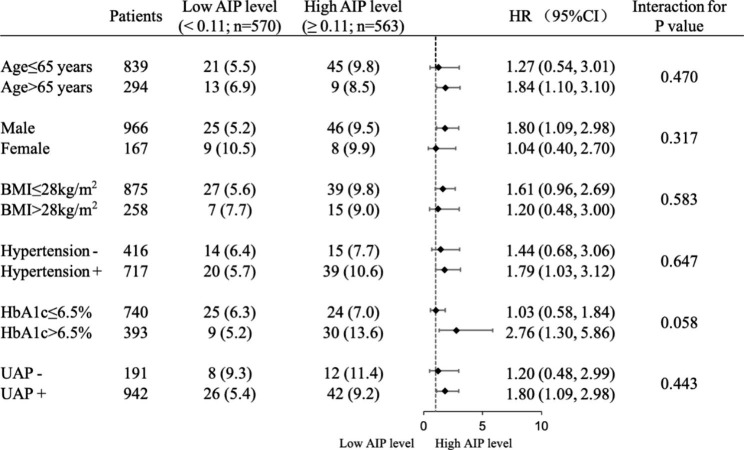
Subgroup analysis for the impact of AIP on the risk of MACCE. AIP, atherogenic index of plasma; BMI, body mass index; CI, confidence interval; HbA1c, glycosylated hemoglobin; HR, hazard ratio; UAP, unstable angina pectoris; MACCE, major adverse cardiovascular and cerebrovascular event

The stratified analyses of the predictive value of AIP on the primary endpoint event according to age, sex, BMI, hypertension, HbA1c, and the type of ACS were presented in Fig. 4. A positive association between AIP and incident MACCE were detected among patients older than 65 years, male, or those with BMI ≤ 28 kg/m^2^, hypertension, HbA1c > 6.5%, or unstable angina. There was no significant interaction between AIP and these subgroups.

**Fig. 5:**
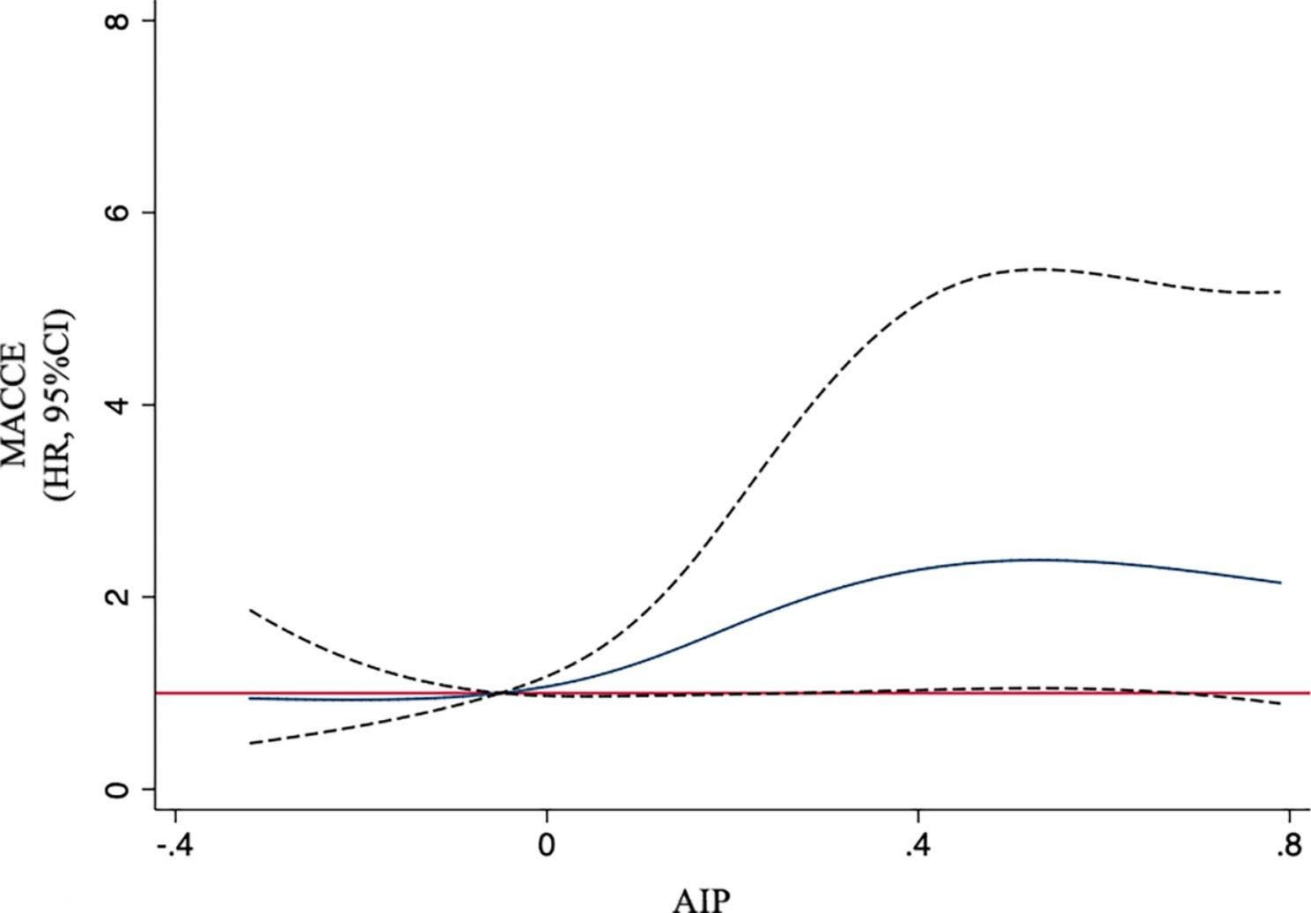
Association of AIP and the risk of MACCE using a multivariable-adjusted restricted cubic spines model. Four knots were used, located at the 5th, 35th, 65th, and 95th percentiles of AIP. Solid blue line represents multivariable adjusted hazard ratio, with dashed black lines showing 95% confidence interval. Analyses were adjusted for sex, age, body mass index, hypertension, dyslipidemia, diabetes mellitus, previous MI, previous stroke, oral hypoglycemic agents, low-density lipoprotein-cholesterol, total cholesterol, glycosylated hemoglobin, and uric acid. AIP, atherogenic index of plasma; CI, confidence interval; HR, hazard ratio; MACCE, major adverse cardiovascular and cerebrovascular event

As shown in Fig. 5, restricted cubic splines revealed a non-linear relationship between AIP and the incidence of MACCE. A positive trend was observed in the incidence of MACCE as AIP increased. The incidence of MACCE ceased to increase beyond an AIP value of 0.50.

## Discussion

In this study, the prognostic significance of AIP was examined in patients with ACS who underwent PCI with LDL-C levels below 1.8mmol/L. The results indicate that patients with high AIP levels had a greater incidence of composite MACCE compared to those with low AIP level, primarily due to an elevated risk of unplanned repeat revascularization. Additionally, the study found that high AIP levels were independently associated with an increased risk of MACCE, even after controlling for potential confounding variables, regardless of whether AIP was treated as a nominal or continuous variable.

Despite adherence to lipid-lowering medication guidelines and achieving recommended LDL-C levels, residual risk of cardiovascular events persists in ACS patients following PCI. This is particularly evident in patients with atherogenic dyslipidemia, characterized by elevated TG levels or low HDL-C levels [4, 18]. This prompts the development of other novel cardiometabolic risk factors to distinguish high-risk populations. Recent research has demonstrated that the atherogenic index of plasma (AIP), a composite indicator based on commonly used lipid parameters TG and HDL-C, is a superior predictor of plasma atherogenicity compared to isolated lipid values [19], and exhibits a strong correlation with an increased incidence of subclinical or symptomatic CAD [20, 21]. Moreover, AIP has been identified as a significant prognosticator of cardiovascular events in CAD patients, regardless of whether they have undergone PCI [11, 13, 22]. It is noteworthy that the aforementioned studies assessed the influence of AIP on cardiovascular prognosis without taking into account the LDL-C level. Moreover, the mean LDL-C level in the study participants remained higher than 2.4 mmol/L, surpassing the recommended threshold of 1.8 mmol/L. These factors may have impacted the predictive value of AIP on cardiovascular events. Furthermore, subgroup analysis examining the association between AIP and cardiovascular outcomes based on LDL-C level yielded inconsistent results. It remains unclear whether AIP retains its predictive value for adverse cardiovascular events in ACS patients who exhibit an LDL-C level below 1.8 mmol/L. Our study aimed to address this knowledge gap and revealed that elevated AIP levels were significantly associated with an increased risk of MACCE in ACS patients, despite well-controlled LDL-C levels. Furthermore, this significant association was primarily driven by an augmented risk of unplanned repeat revascularization. The present study’s results reinforce and extend prior observations by demonstrating that AIP may be utilized to partially account for residual cardiovascular risk and identify individuals who are susceptible to developing MACCE, particularly among those with optimally controlled LDL-C levels. Consequently, interventions aimed at lowering TG levels may provide additional benefits in reducing cardiovascular events in patients who have achieved their LDL-C targets.

While the underlying mechanism behind these findings remains unclear, there are likely multiple plausible explanations. One possible explanation is that atherogenicity may be a significant factor, as AIP is derived from the formula of TG and HDL-C. When plasma TG is higher, LDL-C particle phenotype tend to be smaller, denser, more easily oxidized, and more prone to enter subintima, thereby increasing their atherogenic potential [6, 23, 24]. Previous research has demonstrated a positive correlation between AIP and the particle size of small, dense LDLs, indicating that AIP may serve as a reliable marker of atherogenicity [25–27]. The current investigation yielded evidence that patients exhibiting elevated AIP levels were at a greater risk of MACCE compared to those with lower AIP levels, primarily due to the heightened likelihood of unplanned repeat revascularization. Previous research has demonstrated a positive association between AIP levels and the severity of coronary artery lesions as well as plaque stability [7, 28, 29].

This meant that patients exhibiting elevated AIP levels are more susceptible to accelerated progression and rupture of coronary plaques, thereby elevating the likelihood of unplanned repeat revascularization. Secondly, several studies have demonstrated significant associations between elevated AIP level and insulin resistance, which is associated with an augmented susceptibility to cardiovascular events [30]. Thirdly, patients with a high AIP level were more likely to be obese [31], and manifest a greater incidence of hypertension, diabetes mellitus, and metabolic syndrome [32–34], all of which which are essential players in poorer clinical outcomes following PCI.

There are several limitations that require consideration in the current study. First, due to the observational nature of the study, despite adjusting for potential cardiac risk factors, residual or unmeasured confounding may still exist. Second, the relatively low incidence of events in the present analysis may be attributed to the high use rate of ticagrelor-based dual antiplatelet regimen and low levels of LDL-C. Nevertheless, a positive correlation between AIP and cardiovascular events was still observed. Third, the low rate of MI in the low AIP group may have hindered the detection of significant differences in the risk of MI between groups, and adequately powered prospective cohort studies will be necessary to confirm the relationship of AIP and the risk of MI. Forth, the observed positive correlation between AIP and MACCE was primarily influenced by the rate of unplanned repeat revascularization. However, the current database does not provide details regarding the specific type of revascularization. Fifth, the participants in this study were exclusively Chinese patients, and the generalizability of the findings to other ethnic groups remains uncertain. Sixthly, the investigation solely examined the baseline AIP, and the longitudinal consecutive changes of AIP during the follow-up period were not analyzed. Seventh, the non-linear association between AIP and MACCE did not attain statistical significance due to the limited sample size. Lasty, the information on some lipid-lowering agents and their respective dosages, such as statins, niacin or fibrates, or new hypoglycemic agents, which may have influenced the results, were not available in our database.

## Conclusions

Elevated AIP is independently associated with the risk of MACCE in patients with ACS who have undergone PCI and have LDL-C levels below 1.8mmol/L. The simple index, which can be obtained from a routine lipid profile, may offer supplementary prognostic information for ACS patients with optimally managed LDL-C levels.

## Electronic supplementary material

Below is the link to the electronic supplementary material.

Supplementary Material 1

## Data Availability

The datasets used and/or analyzed in the study are available from the corresponding author upon reasonable request.

## Abbreviations

AIP: atherogenic index of plasma
ACS: acute coronary syndrome
BMI: body mass index
CAD: coronary artery disease
CI: confidence interval
FBG: fasting blood glucose
HbA1c: hemoglobin A1c
HDL-C: high-density lipoprotein cholesterol
HR: hazard ratio
IQR: interquartile range
LDL-C: low-density lipoprotein cholesterol
MACCE: major adverse cardiovascular and cerebrovascular event
MI: myocardial infarction
PCI: percutaneous coronary intervention
TC: total cholesterol
TG: triglyceride
