# Supplementary material for: The predictive value of atherogenic index of plasma for cardiovascular outcomes in patients with acute coronary syndrome undergoing percutaneous coronary intervention with LDL-C below 1.8mmol/L
Source: Cardiovasc Diabetol. 2023 Jun 26;22:150. doi: 10.1186/s12933-023-01888-3 (PMC10294439; doi:10.1186/s12933-023-01888-3)
Supplement: Supplementary file 1 — Supplementary Material 1 [file 12933_2023_1888_MOESM1_ESM.docx]

**Supplemental Material**

**
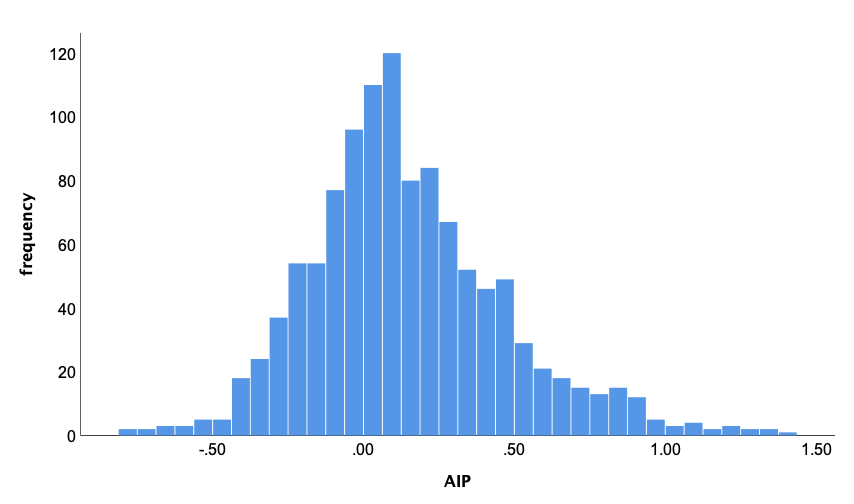
**

**Figure S1.** Frequency histograms of AIP in the overall cohort.

**Table S1. Participant characteristics stratified by low-density lipoprotein cholesterol level**

|  | **LDL< 1.8 mmol/L ( n=1,220)** | **LDL≥1.8mmol/L ( n=2,328)** |  |
| --- | --- | --- | --- |
| **Age (y)** | 58.6±9.5 | 57.8±9.9 | 0.028 |
| **Sex, male** | 1041 (85.3) | 1817 (78.0) | <0.001 |
| **BMI, kg/m^2^** | 25.9±3.0 | 26.2±3.2 | 0.030 |
| **Risk factors** |  |  |  |
| Hypertension | 772 (63.3) | 1434 (61.6) | 0.337 |
| Dyslipidemia | 682 (55.9) | 1505 (64.6) | <0.001 |
| Diabetes mellitus | 525 (43.0) | 837 (36.0) | <0.001 |
| Current smoker | 490 (40.2) | 763 (32.8) | <0.001 |
| **Medical history** |  |  |  |
| Prior MI | 314 (25.7) | 378 (16.2) | <0.001 |
| Prior PCI | 440 (36.1) | 390 (16.8) | <0.001 |
| Prior CABG | 23 (1.9) | 37 (1.6) | 0.571 |
| Prior stroke | 66 (5.4) | 179 (7.7) | 0.012 |
| PAD | 17 (1.4) | 43 (1.8) | 0.354 |
| CKD | 31 (2.5) | 39 (1.7) | 0.111 |
| **ACS type** |  |  | <0.001 |
| STEMI | 1017 (83.3) | 1627 (69.9) |  |
| NSTEMI | 101 (8.3) | 332 (14.3) |  |
| Unstable angina | 102 (8.4) | 369 (15.9) |  |
| **Procedure characteristics** | | |  |
| Lesion complexity |  |  |  |
| Left main lesion | 142 (11.6) | 242 (10.4) | 0.299 |
| Bifurcation lesion | 156 (12.8) | 342 (14.7) | 0.198 |
| Chronic total occlusion | 293 (24.0) | 500 (21.5) | 0.093 |
| Target vessel territory |  |  |  |
| Left main | 118 (9.7) | 162 (7) | 0.005 |
| Left anterior descending artery | 721 (59.1) | 1373 (59) | 0.93 |
| Left circumflex | 322 (26.4) | 626 (26.9) | 0.755 |
| Right coronary artery | 421 (34.5) | 768 (33.0) | 0.374 |
| Multivessel intervention | 354 (29.0) | 603 (25.9) | 0.051 |
| Stent number | 2.0 (1.0, 2.0) | 2.0 (1.0, 2.0) | 0.734 |
| Mean stent diameter, mm | 3±0.4 | 3±0.4 | 0.589 |
| Total stent length, mm | 38.0 (24.0, 62.0) | 38.0 (23.0, 62.0) | 0.804 |
| **Laboratory results** |  |  |  |
| LDL-C, mmol/L | 1.5±0.2 | 2.9±0.8 | <0.001 |
| HDL-C, mmol/L | 1.0±0.3 | 1.1±0.4 | <0.001 |
| TC, mmol/L | 3.1±0.6 | 4.6±1 | <0.001 |
| TG, mmol/L | 1.21 (0.90, 1.76) | 1.41 (1.06, 1.99) | <0.001 |
| FPG, mmol/L | 5.94 (5.24, 7.66) | 5.91 (5.24, 7.52) | 0.769 |
| HbA1c, % | 6.5±1.3 | 6.5±1.3 | 0.223 |
| Creatinine, μmol/L | 76.1±43.4 | 71.8±17.4 | 0.001 |
| Uric acid, μmol/L | 349.8±84.8 | 355.4±88.2 | 0.076 |
| LVEF, % | 62.1±7.2 | 61.1±8.1 | <0.001 |
| **Medications at discharge** |  |  |  |
| Aspirin | 1218 (99.8) | 2321 (99.7) | 0.501 |
| Ticagrelor | 1220 (100.0) | 2328 (100.0) | NA |
| Statin | 1213 (99.4) | 2271 (98.3) | 0.011 |
| Ezetimibe | 138 (11.3) | 429 (18.5) | <0.001 |
| β-Blocker | 681 (55.8) | 1494 (64.2) | <0.001 |
| ACEI/ARB | 655 (53.7) | 1285 (55.2) | 0.395 |
| Calcium-channel antagonist | 351 (28.8) | 653 (28.0) | 0.658 |
| Oral hypoglycemic agents | 272 (22.3) | 306 (13.1) | <0.001 |
| Metformin | 168 (13.8) | 233 (10.0) | 0.001 |
| Alpha‑glucosidase inhibitor | 207 (17) | 244 (10.5) | <0.001 |
| Meglitinide | 43 (3.5) | 88 (3.8) | 0.715 |
| Sulfonylurea | 94 (7.7) | 121 (5.2) | 0.004 |
| Thiazolidinediones | 6 (0.5) | 11 (0.5) | 0.822 |
| DPP-4inhibitor | 10 (0.8) | 9 (0.4) | 0.118 |
| Insulin | 132 (10.8) | 160 (6.9) | <0.001 |

ACEI/ARB, angiotensin converting enzyme inhibitors/angiotensin receptor blockers; AIP, Atherogenic index of plasma; BMI, body mass index; CABG, coronary artery bypass grafting; CKD, chronic kidney disease; CTO, chronic total occlusion; DPP-4, dipeptidyl peptidase-4; FPG, fasting plasma glucose; HbA1c, glycosylated hemoglobin; HDL-C, high density lipoprotein cholesterol; LDL-C, low density lipoprotein cholesterol; LVEF, left ventricular ejection fraction; MI, myocardial infarction; NSTEMI, No ST-segment elevation myocardial infarction; PAD, peripheral arterial disease; PCI, percutaneous coronary intervention; STEMI, ST-segment elevation myocardial infarction; TC, total cholesterol; TG, triglyceride

**Table S2. Participant characteristics stratified by the primary endpoint event**

|  | **Total (n=1,133)** | **No-MACE (n=1045)** | **MACE (n=88)** | **P value** |
| --- | --- | --- | --- | --- |
| **Age (y)** | 58.6±9.5 | 58.6±9.6 | 58.5±9.0 | 0.923 |
| **Sex, male** | 966 (85.3) | 895 (85.6) | 71 (80.7) | 0.207 |
| **BMI, kg/m^2^** | 25.9±3.0 | 25.9±3.0 | 25.7±3.3 | 0.602 |
| **Risk factors, n (%)** | | | | |
| Hypertension | 717 (63.3) | 658 (63.0) | 59 (67.0) | 0.446 |
| Dyslipidemia | 634 (56.0) | 584 (55.9) | 50 (56.8) | 0.954 |
| Diabetes mellitus | 485 (42.8) | 445 (42.6) | 40 (45.5) | 0.601 |
| Current smoker | 457 (40.3) | 419 (40.1) | 38 (43.2) | 0.571 |
| **Medical history, n (%)** | | | | |
| Prior MI | 291 (25.7) | 259 (24.8) | 32 (36.4) | 0.017 |
| Prior PCI | 408 (36.0) | 368 (35.2) | 40 (45.5) | 0.055 |
| Prior CABG | 21 (1.9) | 19 (1.8) | 2 (2.3) | 1.000 |
| Prior stroke | 61 (5.4) | 56 (5.4) | 5 (5.7) | 1.000 |
| PAD | 16 (1.4) | 15 (1.4) | 1 (1.1) | 1.000 |
| CKD | 28 (2.5) | 25 (2.4) | 3 (3.4) | 0.816 |
| **ACS type, n (%)** | | | | 0.079 |
| STEMI | 96 (8.5) | 89 (8.5) | 7 (8.0) |  |
| NSTEMI | 95 (8.4) | 82 (7.8) | 13 (14.8) |  |
| Unstable angina | 942 (83.1) | 874 (83.6) | 68 (77.3) |  |
| **Procedure characteristics** | | | | |
| Lesion complexity, n (%) | | | | |
| Left main lesion | 135 (11.9) | 122 (11.7) | 13 (14.8) | 0.389 |
| Bifurcation lesion | 127 (11.2) | 118 (11.3) | 9 (10.2) | 0.761 |
| Chronic total occlusion | 272 (24.0) | 246 (23.5) | 26 (29.5) | 0.205 |
| Target vessel territory, n (%) | | | | |
| Left main | 94 (8.3) | 87 (8.3) | 7 (8.0) | 0.904 |
| Left anterior descending artery | 667 (58.9) | 629 (60.2) | 38 (43.2) | 0.002 |
| Left circumflex | 297 (26.2) | 275 (26.3) | 22 (25.0) | 0.788 |
| Right coronary artery | 387 (34.2) | 344 (32.9) | 43 (48.9) | 0.002 |
| Multivessel intervention | 326 (28.8) | 303 (29.0) | 23 (26.1) | 0.569 |
| Stent number | 2.0 (1.0, 2.0) | 2.0 (1.0, 2.0) | 2.0 (1.0, 3.0) | 0.222 |
| Mean stent diameter, mm | 3.0±0.4 | 3.0±0.4 | 2.9±0.4 | 0.135 |
| Total stent length, mm | 38.0 (24.0, 62.0) | 38.0 (24.0, 61.0) | 44.5 (24.0, 74.7) | 0.123 |
| **Laboratory results** | | | | |
| LDL-C, mmol/L | 1.5±0.2 | 1.5±0.2 | 1.5±0.2 | 0.423 |
| HDL-C, mmol/L | 1.0±0.2 | 1.0±0.2 | 1.0±0.3 | 0.444 |
| TC, mmol/L | 3.1±0.6 | 3.1±0.6 | 3.2±0.6 | 0.089 |
| TG, mmol/L | 1.2 (0.9, 1.8) | 1.2 (0.9, 1.8) | 1.4 (1.0, 2.1) | 0.009 |
| FPG, mmol/L | 5.7 (5.3, 7.7) | 6.0 (5.3, 7.6) | 6.3 (5.3, 8.7) | 0.234 |
| HbA1c, % | 6.5±1.3 | 6.5±1.3 | 6.7±1.2 | 0.246 |
| Creatinine, μmol/L | 76.1±43.4 | 75.9±44.0 | 78.2±35.2 | 0.644 |
| Uric acid, μmol/L | 349.8±84.8 | 348.8±83.5 | 362.6±98.2 | 0.140 |
| AIP | 0.1 (-0.1, 0.3) | 0.1 (-0.1, 0.3) | 0.2 (0, 0.4) | 0.011 |
| LVEF, % | 62.1±7.0 | 62.2±6.9 | 61.2±8.3 | 0.190 |
| **Medications at discharge, n (%)** | | | | |
| Aspirin | 1131 (99.8) | 1043 (99.8) | 88 (100) | 1.000 |
| Ticagrelor | 1133 (100) | 1145 (100) | 88 (100) | 1.000 |
| Statin | 1126 (99.4) | 1038 (99.3) | 88 (100) | 0.441 |
| Ezetimibe | 81 (7.1) | 76 (7.3) | 5 (5.7) | 0.578 |
| β-Blocker | 632 (55.8) | 576 (55.1) | 56 (63.6) | 0.122 |
| ACEI/ARB | 608 (53.7) | 558 (53.4) | 50 (56.8) | 0.537 |
| Calcium-channel antagonist | 326 (28.8) | 298 (28.5) | 28 (31.8) | 0.511 |
| Oral hypoglycemic agents | 234 (20.7) | 214 (20.5) | 20 (22.7) | 0.617 |
| Metformin | 115 (10.2) | 108 (10.3) | 7 (8.0) | 0.154 |
| Alpha‑glucosidase inhibitor | 142 (12.5) | 127 (12.2) | 15 (17.0) | 0.026 |
| Meglitinide | 22 (1.9) | 20 (1.9) | 2 (2.3) | 0.155 |
| Sulfonylurea | 64 (5.6) | 60 (5.7) | 4 (4.5) | 0.168 |
| Thiazolidinediones | 6 (0.5) | 6 (0.6) | 0 (0) | 0.112 |
| DPP-4 inhibitor | 7 (0.6) | 6 (0.6) | 1 (1.1) | 0.132 |
| Insulin | 83 (7.3) | 75 (7.2) | 8 (9.1) | 0.091 |

ACEI/ARB, angiotensin converting enzyme inhibitors/angiotensin receptor blockers; AIP, Atherogenic index of plasma; BMI, body mass index; CABG, coronary artery bypass grafting; CKD, chronic kidney disease; CTO, chronic total occlusion; DPP-4, dipeptidyl peptidase-4; FPG, fasting plasma glucose; HbA1c, glycosylated hemoglobin; HDL-C, high density lipoprotein cholesterol; LDL-C, low density lipoprotein cholesterol; LVEF, left ventricular ejection fraction; MI, myocardial infarction; NSTEMI, No ST-segment elevation myocardial infarction; PAD, peripheral arterial disease; PCI, percutaneous coronary intervention; STEMI, ST -segment elevation myocardial infarction; TC, total cholesterol; TG, triglyceride
